# Dectin-3 Recognizes Glucuronoxylomannan of *Cryptococcus neoformans* Serotype AD and *Cryptococcus gattii* Serotype B to Initiate Host Defense Against Cryptococcosis

**DOI:** 10.3389/fimmu.2018.01781

**Published:** 2018-08-06

**Authors:** Hua-Rong Huang, Fan Li, Hua Han, Xia Xu, Ning Li, Shunchun Wang, Jin-Fu Xu, Xin-Ming Jia

**Affiliations:** ^1^Shanghai Skin Disease Hospital, Tongji University School of Medicine, Shanghai, China; ^2^Department of Respiratory and Critical Care Medicine, Shanghai Pulmonary Hospital, Tongji University School of Medicine, Shanghai, China; ^3^Institute of Chinese Materia Medica, Shanghai University of Traditional Chinese Medicine, Shanghai, China

**Keywords:** innate immunity, C-type lectin receptor, Dectin-3, *Crytococcus*, glucuronoxylomannan

## Abstract

*Cryptococcus neoformans* and *Cryptococcus gattii* cause life-threatening meningoencephalitis or lung diseases in immunocompetent individuals or immunocompromised ones. *C. neoformans* and *C. gattii* are subdivided into five serotypes based on their capsular glucuronoxylomannan (GXM). *C. neoformans* consists of serotypes A, D, and AD hybrid, and *C. gattii* consists of serotypes B and C. Given structural differences of GXM between *C. neoformans* and *C. gattii*, it remains unclear that how innate immune system recognizes GXM. Here, we report that C-type lectin receptor Dectin-3 (MCL encoded by Clec4d) is a direct receptor for GXMs from *C. neoformans* serotype AD (*C.n*-AD) and *C. gattii* serotype B (*C.g*-B). GXMs from *C.n*-AD and *C.g*-B activated NF-κB and ERK pathways to induce pro-inflammatory cytokine production, whereas it was completely abolished due to deficiency of Dectin-3 or caspase recruitment domain family member 9 (CARD9). Upon pulmonary *C.n*-AD and *C.g*-B infection, Dectin-3- and CARD9-deficient mice were highly susceptible and showed augmented lung injury due to impairment of alveolar macrophage accumulation and killing activities. Our study provides the first biological and genetic evidence demonstrating that Dectin-3 recognizes GXM of *C.n*-AD and *C.g*-B to initiate host defense against cryptococcosis.

## Introduction

The saprophytic, encapsulated fungal pathogens *Cryptococcus neoformans* and *Cryptococcus gattii* can cause life-threatening meningoencephalitis and pneumonia in both immunocompromised and immunocompetent individuals, which is called cryptococcosis (1). *C. neoformans* has been classified into three serotypes including A, D, and AD hybrid whereas serotypes B and C have been recognized as a separate species called *C. gattii* based on antigenic differences in the polysaccharide capsules of the fungus (2–4). *C. neoformans* serotype A (*C.n*-A) is the most clinically prevalent in immunocompromised individuals, including HIV patients, renal transplant recipients, and those undergoing immunosuppressive therapy (5), whereas *C. neoformans* serotype D (*C.n*-D) is mostly found in Europe but has a sporadic global distribution (6). A recent survey in Europe revealed that 19% of human infections are caused by *C. neoformans* serotype AD (*C.n*-AD), which is a hybrid of serotype A and D strains (7). In contrast, infection by *C. gattii* including serotype B and C (*C.g*-B and *C.g*-C) is much less common in immunocompromised patients but is thought to be more virulent than *C. neoformans* and causes disseminated infections even in healthy hosts (8). Previously, it was thought that *C. gattii* infections were restricted to tropical and subtropical regions (9), but the emergence of the outbreak events due to *C. gattii* infections in temperate areas of North America suggest a more global distribution of this yeast (10, 11).

Both *C*. *neoformans* and *C. gattii* are found ubiquitously in soil and other niches, and inhalation of yeast or desiccated basidiospores into the lungs is extremely common, causing about 70% of children with pulmonary infections in urban environments (12). Host immune cells including alveolar macrophages (AMs) and dendritic cells (DCs) recognize pathogen-associated molecular patterns *via* pattern-recognition receptors (PRRs), which elicit the host defense response. C-type lectin receptors (CLRs), a PRR-recognizing PAMP composed of polysaccharides, have garnered the attention of many investigators in the study of host defense against fungal infection (13, 14). The genetic deficiency of Dectin-1, a representative CLR recognizing β1,3-glucans of *Candida albicans* yeast and *Aspergillus fumigatus* conidia (15, 16), did not influence the clearance of *C.n*-A pulmonary infections (17). Dectin-2 is known to recognize α-1,2-mannans of *C. albicans* and *A. fumigatus* hyphae, lipophilic and hydrophilic components of Malassezia, mannose-capped lipoarabinomannan of *Mycobacterium tuberculosis* and unknown components of non-capsular *C.n*-A to trigger the production of various cytokines and chemokines, including pro-inflammatory Th1, Th17, and also Th2 cytokines (18–22). Our earlier studies show that Dectin-3 (also called CLECSF8, MCL, or Clec4d) can recognize α-1,2-mannans of *C. albicans* hyphae and trehalose 6,6’-dimycolate of *M. tuberculosis* (23, 24). However, a recent study shows that Dectin-3 is dispensable for mediating protective immune responses against pulmonary *C.n*-A infection (25). The genetic defect of the caspase recruitment domain family member 9 (CARD9), an adaptor protein that operates downstream of CLRs for activating NF-κB and extracellular signal-regulated protein kinase (ERK) pathways (26, 27), confers susceptibilities to pulmonary *C.n*-A infection probably due to the reduced accumulation of IFN-γ-expressing NK and memory T cells (28).

Cryptococcal capsule is composed primarily of glucuronoxylomannan (GXM), which comprises more than 90% of the capsule’s polysaccharide mass (29). The typical GXM consists of a linear (1→3)-α-d-mannopyranan bearing β-d-xylopyranosyl (Xylp), β-d-glucopyranosyluronic acid, and 6-O-acetyl substituents (30–32). The disposition of the O-acetyl substituents is the major determinant of the antigenic activity observed among GXMs obtained from all serotypes (A, B, C, D, and AD) (33). The ability of GXM to activate the toll-like receptor (TLR)-mediated innate immune response has been reported in several studies (34–36). In detail, GXM from *C.n*-A activates TLR4-mediated intracellular signaling (34), but its contribution to the global innate response against *C. neoformans* infections is limited (37, 38). GXM from *C.n*-A can also interact with TLR2 (34), which is believed to influence the response to cryptococcal infection (35). A recent study shows that GXM from five cryptococcal serotypes were differentially recognized by TLR2/TLR1 and TLR2/TLR6 heterodimers (36). Overall, most studies on the immunological functions of GXM have focused on the polysaccharide fractions from *C.n*-A isolates. Given the structural differences of GXM among the five serotypes, it also remains unknown that how host innate immune system differentially recognizes these GXMs.

In the present study, we show that Dectin-3 is a direct receptor for capsular GXM from *C.g*-B and *C.n*-AD, but not *C.n*-A, *C.n*-D, and *C.g*-C. Furthermore, we demonstrate that Dectin-3 is essential for GXM-induced inflammatory responses through activating NF-κB and ERK pathways. Therefore, Dectin-3-deficient mice are highly sensitive to pulmonary *C. g*-B and *C.n*-AD infections due to impairment of alveolar macrophage activation.

## Results

### Dectin-3 Recognizes the Capsule of *C.g*-B to Induce NF-κB and ERK-Mediated Pro-Inflammation Responses

**Figure 1:**
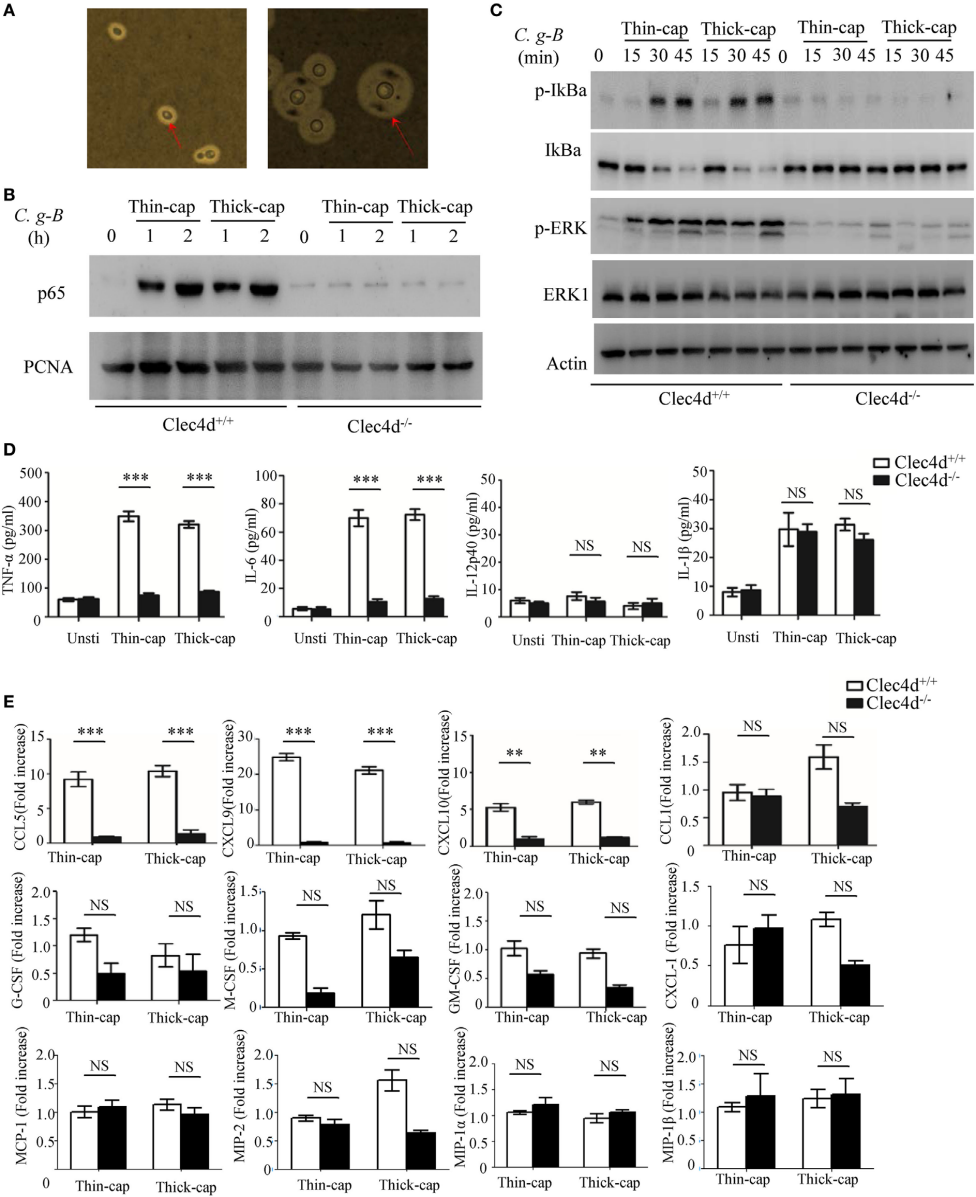
Dectin-3 is essential for *Cryptococcus gattii*-B-induced NF-κB and ERK-mediated pro-inflammation responses. **(A)** *C.g-*B strain ATCC32609 was cultured with thin-capsule form in yeast extract peptone dextrose medium at 30°C and induced into thick-capsule form in RPMI-1640 medium plus 10% FBS at 37°C with 5% CO_2_. Arrows indicate capsule of *C.g-*B strain. **(B,C)** Nuclear protein **(B)** and total protein phosphorylation **(C)** levels in bone marrow-derived macrophages (BMDMs) from wild-type (WT) and Dectin-3(Clec4d)-deficient mice, stimulated with thin- and thick-capsulated *C.g*-B strain (MOI = 5) for indicated time. **(D)** Enzyme-linked immune-sorbent assay results for indicated cytokines in WT and Dectin-3-deficient BMDMs, stimulated with thin- and thick-capsulated *C.g*-B strain (MOI = 5) for 16 h. Data are means ± SD of triplicate wells and are representative of three independent experiments; NS = no significant difference, **p < 0.01 and ***p < 0.001. **(E)** mRNA expression levels of indicated chemokines in WT and Dectin-3-deficient BMDMs, stimulated with thin- and thick-capsulated *C.g*-B strain (MOI = 5) for 3 h.

In nature and under laboratory conditions, the capsule of *Cryptococcus* strains is relatively thin but can be mature to become thick during mammalian infection (39). In this study, *C.g*-B strain ATCC32609 was prepared as thin- and thick-capsulated suspensions as described (39) and capsule induction was confirmed by India ink staining (Figure 1A). To explore whether Dectin-3 is required for *C.g*-B induced pro-inflammation responses, we stimulated bone marrow-derived macrophages (BMDMs) from wild-type (WT, *Clec4d^+/+^*) and Dectin-3-deficient (*Clec^−/−^*) mice with thin- and thick-capsulated *C.g*-B, and found that both thin- and thick-capsulated *C.g*-B strongly induced nuclear translocation of NF-κB (p65 subunit) (Figure 1B), together with IκBα phosphorylation and degradation (Figure 1C). In contrast, Dectin-3 deficiency completely impaired the *C.g*-B-induced NF-κB nuclear translocation (Figure 1B) and IκBα phosphorylation and degradation (Figure 1C). Moreover, both thin- and thick-capsulated *C.g*-B triggered sustained phosphorylation of ERK in WT BMDMs (Figure 1C), but Dectin-3 deficiency dramatically impaired *C.g*-B-induced ERK activation (Figure 1C). Together, these results indicate that Dectin-3 is critical for *C.g*-B-induced activation of NF-κB and ERK pathways. Furthermore, we found that secretion of pro-inflammation cytokines including TNF-α and IL-6 were increased in WT BMDMs when stimulated with thin- and thick-capsulated *C.g*-B, whereas Dectin-3 deficiency significantly impaired these responses (Figure 1D). However, *C.g*-B-induced levels of IL-12p40 and IL-1β were comparable in WT and Dectin-3-deficient BMDMs (Figure 1D). These results suggest that Dectin-3 is essential for *C.g*-B-induced NF-κB and ERK-mediated pro-inflammation responses.

During cryptococcal infection, various chemokines are produced by alveolar monocytes to attract inflammatory cells and specific leukocyte subsets from blood to the site of infection. Here, we found that Dectin-3 deficiency in BMDMs strikingly impaired *C.g*-B-induced expression of CCL5/RANTES and of C-X-C motif chemokine ligand 9 (CXCL9) and CXCL10 (Figure 1E), which can attract activated T cells (particularly Th1 cells), DCs, and monocytes by binding to specific CC chemokine receptors (CCR) on these cells, including CCR5 and CXCR3 (28). However, there were slight influences of Dectin-3 deficiency on *C.g*-B-induced expression of CCL1, CXCL-1, macrophage inflammatory protein-1α, MIP-1β, MIP-2, monocyte chemotactic protein-1, granulocyte-macrophage colony-stimulating factor (GM-CSF), G-CSF, and M-CSF in BMDMs (Figure 1E). These results suggest that Dectin-3 is also essential for *C.g*-B-induced NF-κB and ERK-mediated chemokine productions, resulting in the formation of granulomas and activation of adaptive immunity.

### Dectin-3 Can Also Recognize the Capsule of *C.n*-AD to Induce Pro-Inflammation Responses

**Figure 2:**
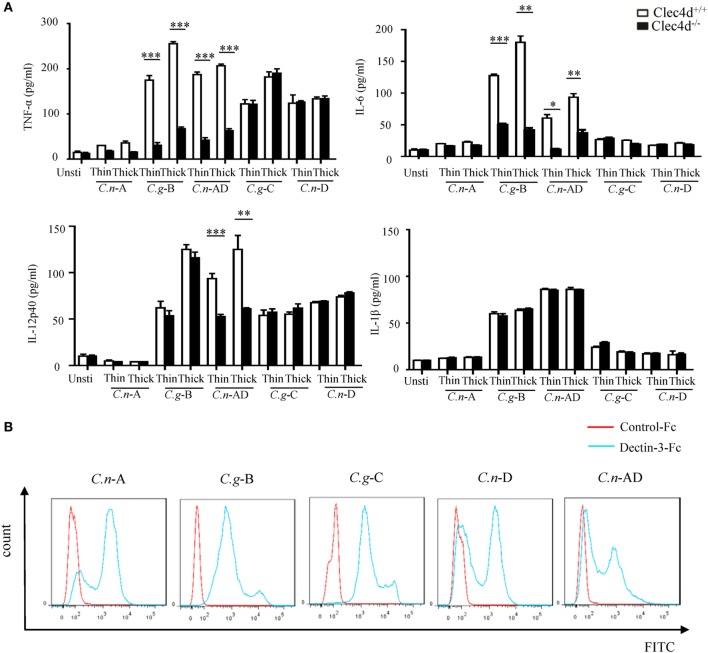
Dectin-3 is also critical for NF-κB and ERK-mediated pro-inflammation responses induced by *Cryptococcus neoformans*-AD. **(A)** Enzyme-linked immune-sorbent assay results for indicated cytokines in wild-type and Dectin-3-deficient bone marrow-derived macrophages, stimulated with thin- and thick-capsulated *C.n*-A strain H99, *C.g-*B strain WM179, *C.n-*AD strain WM628, *C.g-*C strain NIH312, and *C.n-*D strain WM629 (MOI = 5) for 16 h. Data are means ± SD of triplicate wells and are representative of three independent experiments; **p < 0.01 and ***p < 0.001. **(B)** Fluorescence-activated cell-sorting assay of the binding of human Dectin-3-Fc fusion protein to *C.n-*A strain H99, *C.g-*B strain WM179, *C.n-*AD strain WM628, *C.g-*C strain NIH312, and *C.n-*D strain WM629 compared to Control-Fc.

A recent study shows that Dectin-3 is not required for mediating protective immune responses against pulmonary *C.n*-A infection (25). Here, we found that stimulation with *C.n*-A failed to induce the production of pro-inflammation cytokines, including TNF-α, IL-6, IL-12p40, and IL-1β in WT and Dectin-3-deficient BMDMs (Figure 2A). However, stimulation with *C.n*-AD, *C.g*-C, and *C.n*-D could induce large amounts of pro-inflammation cytokines, including TNF-α, IL-6, and IL-12p40 in WT BMDMs (Figure 2A). Of note, Dectin-3 deficiency significantly impaired the production of TNF-α, IL-6, and IL-12p40 in BMDMs when stimulated with both thin- and thick-capsulated *C.n*-AD, but not *C.g*-C and *C.n*-D (Figure 2A). As controls, Dectin-3 deficiency only affected TNF-α and IL-6 secretion in BMDMs when stimulated with another *C.g*-B strain WM179 (Figure 2A). Unexpectedly, Dectin-3 deficiency had no influence on the IL-1β production when stimulated with *C.n*-AD and *C.g*-B (Figure 2A). These results suggest that Dectin-3 is also critical for mediating pro-inflammation responses induced by *C.n*-AD, but not *C.n-*A, *C.n-*D, or *C.g*-C.

To explore whether Dectin-3 can bind the surface capsule of *Cryptococcus* strains, we performed a fluorescence-activated cell-sorting (FACS) screening assay using soluble receptor fusion protein composed of the extracellular portion of Dectin-3 fused with the Fc fragment of human IgG1 antibody (Figure S1 in Supplementary Material). As shown in Figure 2B, Dectin-3-Fc fusion protein could bind all five serotypes of *Cryptococcus* strains compared to control-Fc. Together, these results indicate that although Dectin-3 can recognize all the serotypes of *Cryptococcus* strains, only *C.n*-AD and *C.g-*B can initiate the pro-inflammation responses dependent of Dectin-3 recognition.

### Dectin-3 Directly Recognizes GXM From *Cryptococcus* Strains

**Figure 3:**
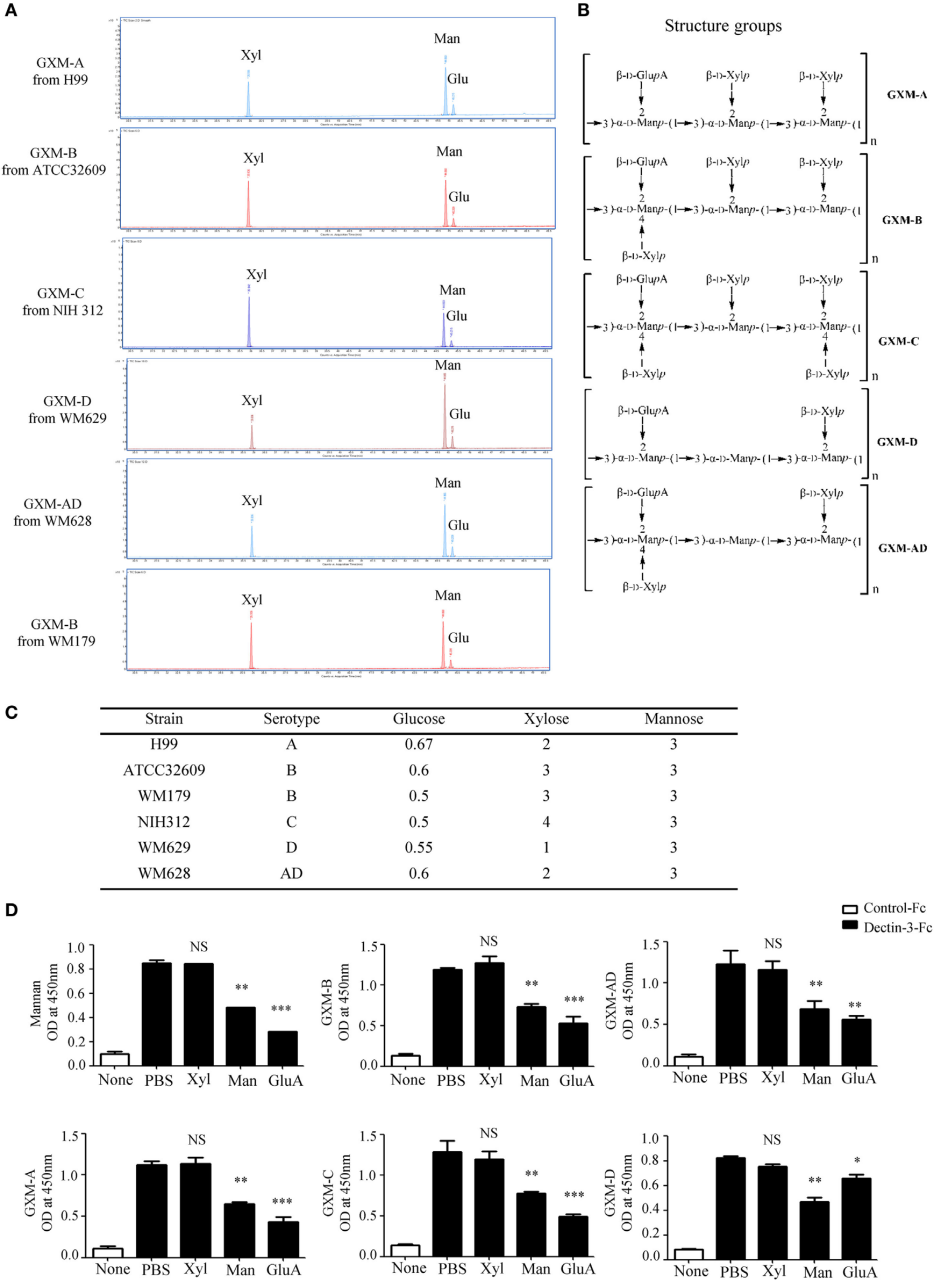
Dectin-3 directly recognizes GXM from *Cryptococcus* strains. **(A)** Monosaccharide assay of GXM from *C.n-*A strain H99, *C.g-*B strain ATCC32609, *C.g-*B strain WM179, *C.n-*AD strain WM628, *C.g-*C strain NIH312, and *C.n-*D strain WM629 by gas chromatography–mass spectrometry (GC–MS) after uronic acid reduction treatment. **(B)** Predicted structures of GXM from five serotypes of *Cryptococcus* strains. **(C)** Analysis of monosaccharide composition of GXM. **(D)** Enzyme-linked immune-sorbent assay binding assay of human control-Fc and Dectin-3-Fc fusion protein to five serotypes of GXM and mannan. Dectin-3-Fc fusion protein was saturated with mannan/xylose/glucuronic acid, respectively. Data are means ± SD of triplicate wells and are representative of three independent experiments; NS = no significant difference, *p < 0.05, **p < 0.01, and ***p < 0.001.

It has been well-documented that cryptococcal capsule is primarily composed of GXM (29). We extracted extracellular polysaccharides (EPS) from *C.n*-A, *C.g*-B, *C.g*-C, *C.n*-D, and *C.n*-AD as previously reported and the extract procedure was shown in Figure S2A in Supplementary Material. The extracted EPS fraction is a mixture of several polysaccharides, so we performed anion column chromatography to obtain major components for exploring their structural differences. Since the GXMs from *Cryptococcus* strains contained uronic acid (32, 40), we performed reduction reactions to change glucuronic acid (GluA) into glucose (Glu) of EPS for GC–MS analysis (Figure S2B in Supplementary Material; Figure 3A). Monosaccharide composition analysis showed that the EPS from *C.n*-A, *C.g*-B, *C.g*-C, *C.n*-D, and *C.n*-AD mainly contained glucose (Glu), xylose (Xyl), and mannose (Man) (Figure S2B in Supplementary Material; Figure 3A), indicating that EPS are primarily composed of GXM, a major cryptococcal capsule component. It has been reported that GXMs from *Cryptococcus* strains are all comprised of a core repeating unit to which (1→2)-linked and (1→4)-linked β-d-Xyl units are added in increments of one to four residues (Figure 3B) (32, 40). GXMs from *C.n*-A and *C.n*-D are mainly substituted with Xyl at C-2, whereas GXMs from *C.g*-B, *C.g*-C, and *C.n*-AD are substituted at C-2 and at C-4 (Figure 3B). Analytical data showed that molar ratios of Glu/Xyl/Man in *C.n*-A, *C.g*-B, *C.g*-C, *C.n*-D, and *C.n*-AD were calculated as 0.67:2:3, 0.6:3:3, 0.5:4:3, 0.55:1:3, and 0.58:2:3, respectively (Figure S2C in Supplementary Material; Figure 3C), which demonstrated that all serotypes of GXM have the same repeating unit composed of mannan and GluA as main chain whereas the quantity and position of xylose residue is the major structural difference of GXMs among different serotypes.

To determine whether Dectin-3 can directly recognize GXM from *Cryptococcus* strains, we used an enzyme-linked immune-sorbent assay (ELISA)-based method to examine the direct binding of plate-coated GXM extracted from *Cryptococcus* strains with Fc-fusion Dectin-3 protein, which were known to be bond with plate-coated α-1,2-mannan. We found that Dectin-3 could bind GXM from all five serotypes of *Cryptococcus* strains (Figure 3D). Furthermore, to determine which group does Dectin-3 directly recognizes, we used xylose, mannan, and GluA, to saturate Dectin-3-Fc fusion protein separately before ELISA binding assay. It showed that both mannan and GluA instead of xylose saturation could block the binding of Dectin-3 to GXM (Figure 3D). Together, these data indicate that Dectin-3 can directly recognize GXM from all five serotypes of *Cryptococcus* strains. The general repeating unit composed of mannan and GluA determines its binding with Dectin-3.

### Dectin-3 Recognizes GXM From *C.g*-B and *C.n*-AD to Induce NF-κB- and ERK-Mediated Pro-Inflammation Responses

**Figure 4:**
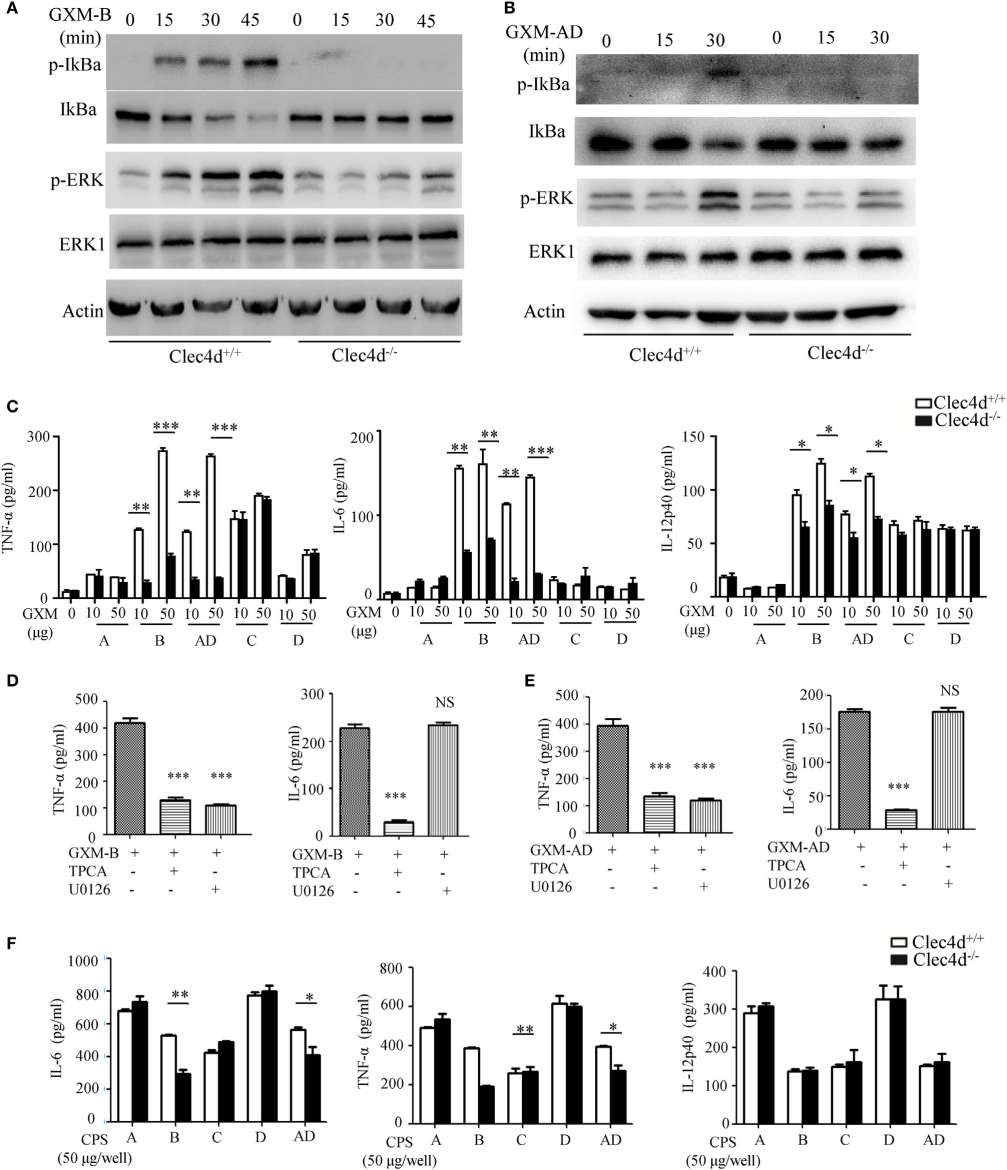
GXMs from *Cryptococcus gattii*-B and *Cryptococcus neoformans*-AD induce NF-κB- and ERK-mediated pro-inflammation responses through Dectin-3. **(A,B)** Protein phosphorylation levels in bone marrow-derived macrophages (BMDMs) from wild-type (WT) and Dectin-3 (Clec4d)-deficient mice, stimulated with plate-coated 50 μg/well GXM from *C.g-*B strain ATCC32609 [GXM-B, **(A)**] or *C.n-*AD strain WM628 [GXM-AD, **(B)**] for indicated time. **(C)** Enzyme-linked immune-sorbent assay (ELISA) results for indicated cytokines in WT and Dectin-3-deficient BMDMs, stimulated with plate-coated GXM extracted from *C.n*-A strain H99, *C.g-*B strain ATCC32609, *C.n-*AD strain WM628, *C.g-*C strain NIH312, and *C.n-*D strain WM629 for 16 h. **(D,E)** ELISA results for indicated cytokines in WT BMDMs, pretreated with 10 µM TPCA (p65 inhibitor) or 10 µM U0126 (ERK inhibitor) and stimulated with plate-coated GXM-B **(D)** or GXM-AD **(E)** for 16 h. **(F)** ELISA results for indicated cytokines in WT and Dectin-3-deficient BMDMs, stimulated with plate-coated CPS (50 μg/well) of five serotypes for 16 h. Data are means ± SD of triplicate wells and are representative of three independent experiments; NS = no significant difference, *p < 0.05, **p < 0.01, and ***p < 0.001.

To further determine whether Dectin-3 recognizes GXM from *C.g*-B and *C.n*-AD to elicit pro-inflammation responses, we stimulated WT and Dectin-3-deficent BMDMs with plate-coated GXM extracted from *C.g-*B (GXM-B) and *C.n*-AD (GXM-AD). We found that stimulation with plate-coated GXM-B and GXM-AD induced IκBα phosphorylation and degradation and ERK phosphorylation (Figures 4A,B). In contrast, Dectin-3 deficiency in BMDMs strikingly impaired GXM-B or GXM-AD induced activation of NF-κB and ERK pathways (Figures 4A,B). Furthermore, we found that only stimulation with plate-coated GXM-B and GXM-AD potently induced the secretion of pro-inflammation cytokines, including TNF-α, IL-6, and IL-12p40, whereas Dectin-3 deficiency significantly impaired these responses (Figure 4C). We further examined the effect of inhibiting NF-κB or ERK signaling on pro-inflammation cytokine production by WT BMDMs. We found that treatment with TPCA, a specific inhibitor for NF-κB activation, significantly suppressed TNFα and IL-6 production by WT BMDMs when stimulated with plate-coated GXM extracted from either *C.g-*B or *C.n*-AD (Figures 4D,E). However, treatment with U0126, an inhibitor for ERK activation, only blocked TNFα production and had no inhibitory effect on GXM-induced IL-6 production (Figures 4D,E). To verify the pro-inflammatory response to GXM from EPS, we also used capsular polysaccharides (CPS) from five serotypes of *Cryptococcus* strains to stimulate BMDMs. It was shown that the production of IL-6 and TNF-α induced by CPS-B and CPS-AD was dependent of Dectin-3 recognition, which was consistent with the effects of GXM from EPS (Figure 4F). Thus, these data suggest that Dectin-3 recognizes GXM from *C.g*-B and *C.n*-AD to trigger NF-κB- and ERK-mediated pro-inflammation responses.

### Dectin-3-Deficient Mice Are Highly Susceptible to Pulmonary Infection With *C.g*-B and *C.n*-AD

**Figure 5:**
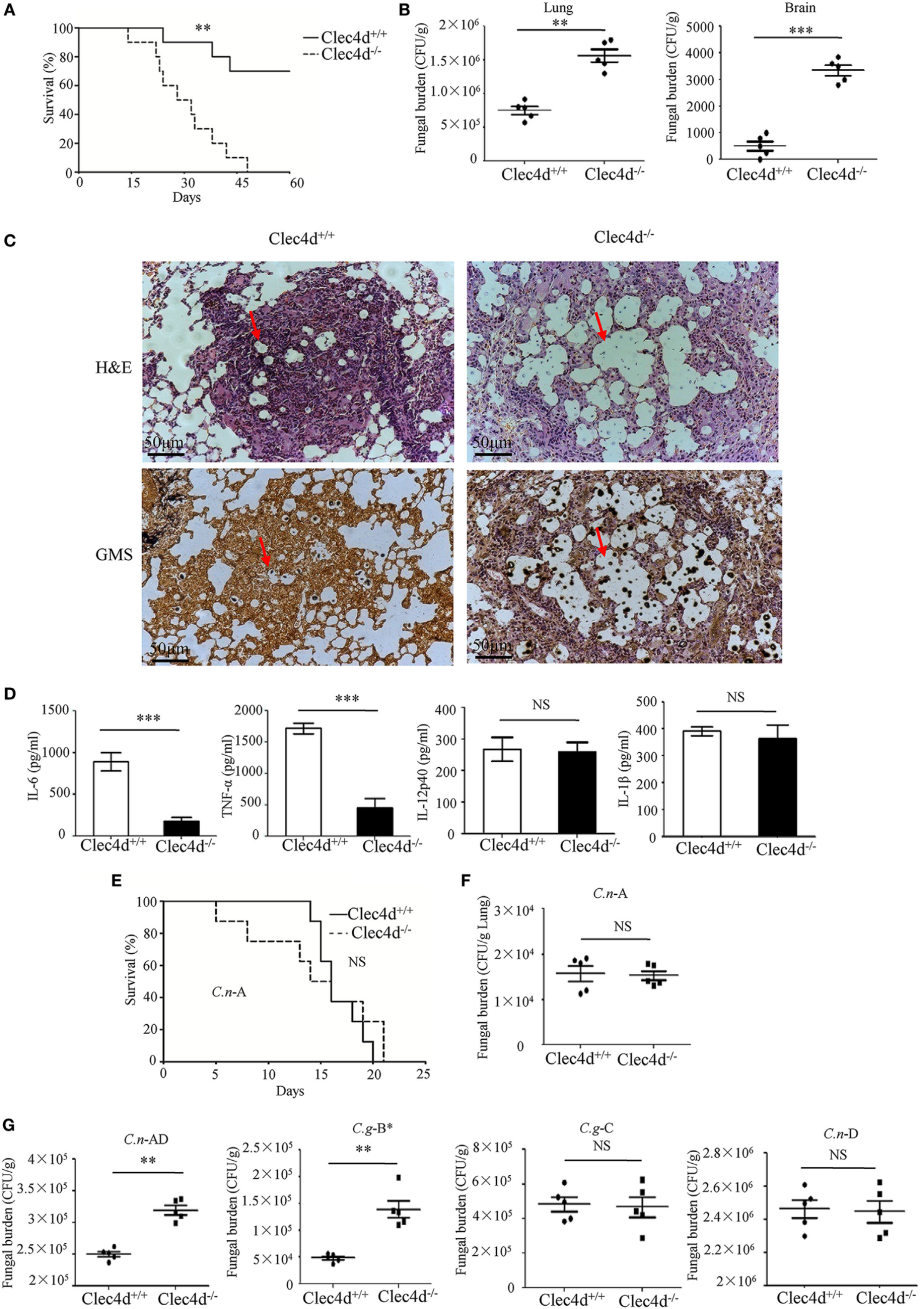
Dectin-3-deficient-mice are highly susceptible to pulmonary infection with *Cryptococcus gattii*-B and *Cryptococcus neoformans*-AD. **(A)** Survival curves of wild-type (WT) and Dectin-3-deficient (Clec4d) mice (*n* = 10 for each group) after intratracheal infection with 1 × 10^6^ CFU of *C.g-*B strain ATCC32609. **(B)** CFU assays of lung and brain of WT and Dectin-3-deficient mice infected intratracheally with 1 × 10^5^ CFU of *C.g-*B on day 3 after infection. **(C)** Histopathology was analyzed with hematoxylin and eosin (H&E) and Gomori’s methenamine silver staining on day 14 after intratracheal infection with 1 × 10^6^ CFU of *C.g-*B. **(D)** Enzyme-linked immune-sorbent assay results of TNF-a, IL-6, IL-12p40, and IL-1β in WT and Dectin-3-deficient mice lung homogenate on day 1 after intratracheal infection with 1 × 10^5^ CFU of *C.g-*B. **(E)** Survival curves of WT and Dectin-3-deficient mice (*n* = 10 for each group) after intratracheal infection with 1 × 10^6^ CFU of *C.n-*A strain H99. **(F)** Lung CFU assay of WT and Dectin-3-deficient mice on day 3 after infection with 1 × 10^5^ CFU of *C.n-*A. **(G)** Lung CFU assays of WT and Dectin-3-deficient mice on day 3 after intratracheal infection with 1 × 10^5^ CFU of *C.n-*AD strain WM628, *C.g-*B strain WM179, *C.g-*C strain NIH312, or *C.n-*D strain WM629, respectively. Data are means ± SD of triplicate wells and are representative of three independent experiments; **p < 0.01 and ***p < 0.001.

To provide genetic evidence that Dectin-3 plays a critical role against experimental pulmonary cryptococcosis, we explored the overall impact of Dectin-3 deficiency during an experimental pulmonary infection with *C.g*-B and *C.n*-AD in mice. WT and Dectin-3-deficient mice received an intratracheal inoculation with *C.g*-B strain ATCC32609. Survival was monitored for greater than 60 days post-inoculation, while pulmonary fungal burden was evaluated in a separate group of infected mice at select time points post-inoculation (Figures 5A,B). We found that all infected Dectin-3-deficient mice died during 45 days, whereas about 70% infected WT mice survived for more than 65 days (*p* < 0.01, Figure 5A). Consistently, the burdens of *C.g*-B in lung and brain on day 3 after infection were significantly higher in Dectin-3-deficient mice than in WT mice (*p* < 0.01 and *p* < 0.001, respectively, Figure 5B). We then conducted a histological analysis of the lungs on day 14 after infection. Massive multiplication of yeast cells with poor granulomatous responses was observed in the alveolar spaces of Dectin-3-deficient mice, whereas WT mice showed a seldom number of yeast cells, which were mostly encapsulated in the granulomatous tissues (Figure 5C). We also detected the significant reductions of pro-inflammatory cytokine including TNF-α and IL-6 in the lungs of Dectin-3-deficient mice than those in WT mice (Figure 5D). However, the amount of IL-12p40 and IL-1β was not significantly decreased in the lungs of Dectin-3-deficient mice (Figure 5D). These results indicate that Dectin-3-mediated signaling is involved in the elimination of pulmonary *C.g*-B infection and induction of a protective inflammatory response.

Consistent with the results in the previous study (25), WT and Dectin-3-deficient mice showed an equivalent susceptibility to pulmonary *C.n*-A strain H99 infection (Figure 5E). Also, no significant differences were observed in the pulmonary fungal burdens of Dectin-3-deficient mice compared to WT mice on day 3 after infection with *C.n*-A strain H99 (Figure 5F). These results indicate that Dectin-3 is not required for host defense against pulmonary *C.n*-A infection. Moreover, Dectin-3-deficient mice exhibited higher fungal burdens in lungs than WT mice after intratracheal infection with *C.n-*AD strain WM628 and *C.g-*B strain WM179 (*p* < 0.01, Figure 5G). However, there were no differences in lung fungal burdens between WT and Dectin-3-deficient mice when infected with *C.g-*C strain NIH312 and *C.n-*D strain WM629 (Figure 5G). Together, these results demonstrate that Dectin-3-deficient mice are highly susceptible to pulmonary *C.g*-B and *C.n-*AD infections.

### Dectin-3 Is Critical for Activation of AMs After Pulmonary *C.g*-B and *C.n*-AD Infection

**Figure 6:**
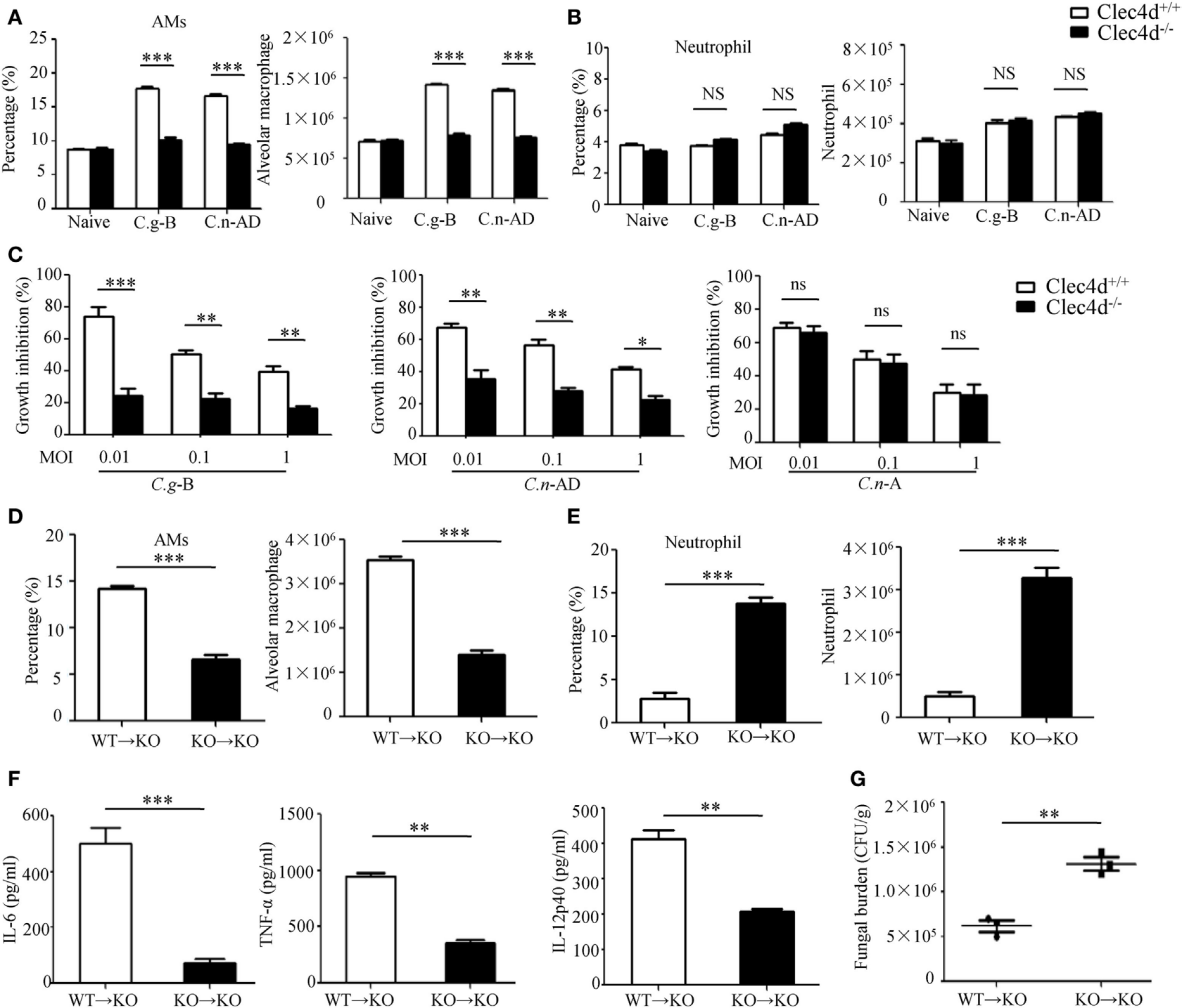
Dectin-3 is critical for activation of alveolar macrophages (AMs) after pulmonary *Cryptococcus gattii*-B and *Cryptococcus neoformans*-AD infection. **(A,B)** AM [CD11c^+^SiglecF^+^, **(A)**] and neutrophil [CD11b^+^Ly6G^+^, **(B)**] counts in lungs of wild-type (WT) and Dectin-3-deficient mice on day 1 after intratracheal infection with *C.g***-**B strain ATCC32609 or *C.n*-AD strain WM628. **(C)** Killing activity of AM sorted from lungs of WT and Dectin-3-deficient mice. *C.g*-B strain ATCC32609, *C.n*-AD strain WM628, or *C.n*-A strain H99 (MOI = 0.01, 0.1, and 1) was co-cultured with or without sorted AM for 6 h and the growth inhibition by AM was calculated. **(D,E)** AM [CD11c^+^SiglecF^+^, **(D)**] and neutrophil [CD11b^+^Ly6G^+^, **(E)**] counts in lungs of Dectin-3-deficient mice, which received intravenous adopt transfer of AMs (5 × 10^5^ cells/mouse) sorted from WT or Dectin-3-deficient mice before infected with 1 × 10^5^ CFU of *C.g-*B strain ATCC32609. **(F)** ELISA results of TNF-a, IL-6, and IL-12p40 in lung homogenate of Dectin-3-deficient mice, receiving intravenous adopt transfer of AM, on day 1 post infection. **(G)** Lung CFU assays of Dectin-3-deficient mice, receiving intravenous adopt transfer of AM, on day 3 post infection. *p < 0.05, **p < 0.01, and ***p < 0.001.

It has been well-documented that AMs constitute the first line of host defense against pulmonary *Cryptococcus* infections and the subsequent inflammatory response, resulting in an influx of neutrophils and monocytes, affords a second line of defense (41). To explore whether Dectin-3 is required for AM and neutrophil accumulation at the early stage of pulmonary infection with *C.g*-B and *C.n*-AD, we determined cellular composition of lungs and found that infected WT mice with *C.g*-B and *C.n*-AD on day 1 showed a prominent AM-biased response, while Dectin-3 deficiency significantly impaired AM accumulation in lungs (*p* < 0.001, Figure 6A; Figure S3A in Supplementary Material). However, either pulmonary *Cryptococcus* infection or Dectin-3 deficiency had no influences on neutrophil accumulation in lungs at the early stage (Figure 6B; Figure S3B in Supplementary Material). Furthermore, we found that AMs sorted from naïve WT mice had high killing activities against *C.n*-A, *C.n*-AD, and *C.g*-B in a dose-dependent manner, whereas Dectin-3 deficiency significantly reduced killing activities of AMs against *C.n*-AD and *C.g*-B, but not *C.n*-A (Figure 6C). These data suggest that Dectin-3 controls AM accumulation and activities after pulmonary infection with *C.g*-B and *C.n*-AD.

To further examine roles of Dectin-3 in AM-mediated host defense against pulmonary infection with *C.g*-B, we performed an adoptive transferring of equal numbers of AMs between WT and Dectin-3-deficient (KO) mice (Figure S3C in Supplementary Material). We found that transferred AMs sorted from WT mice into KO mice could significantly increase AM accumulation and protein levels of pro-inflammation cytokines, including TNF-α, IL-6, and IL-12p40 in lung on day 1 after pulmonary infection with *C.g*-B (Figures 6D,F). Furthermore, the transfer of WT AMs into KO mice significantly reduced fungal burdens in lungs after intratracheal infection with *C.g*-B (*p* < 0.01, Figure 6G). In contrast, transferred AMs sorted from KO mice into KO mice had no any influences on AM accumulation, but significantly increased neutrophil accumulation in lungs (Figures 6D,E). These data confirm that Dectin-3 is critical for accumulation and activities of AM against pulmonary *C.g*-B infection.

### CARD9 Is Critical for Pro-Inflammation Responses Induced by GXMs

**Figure 7:**
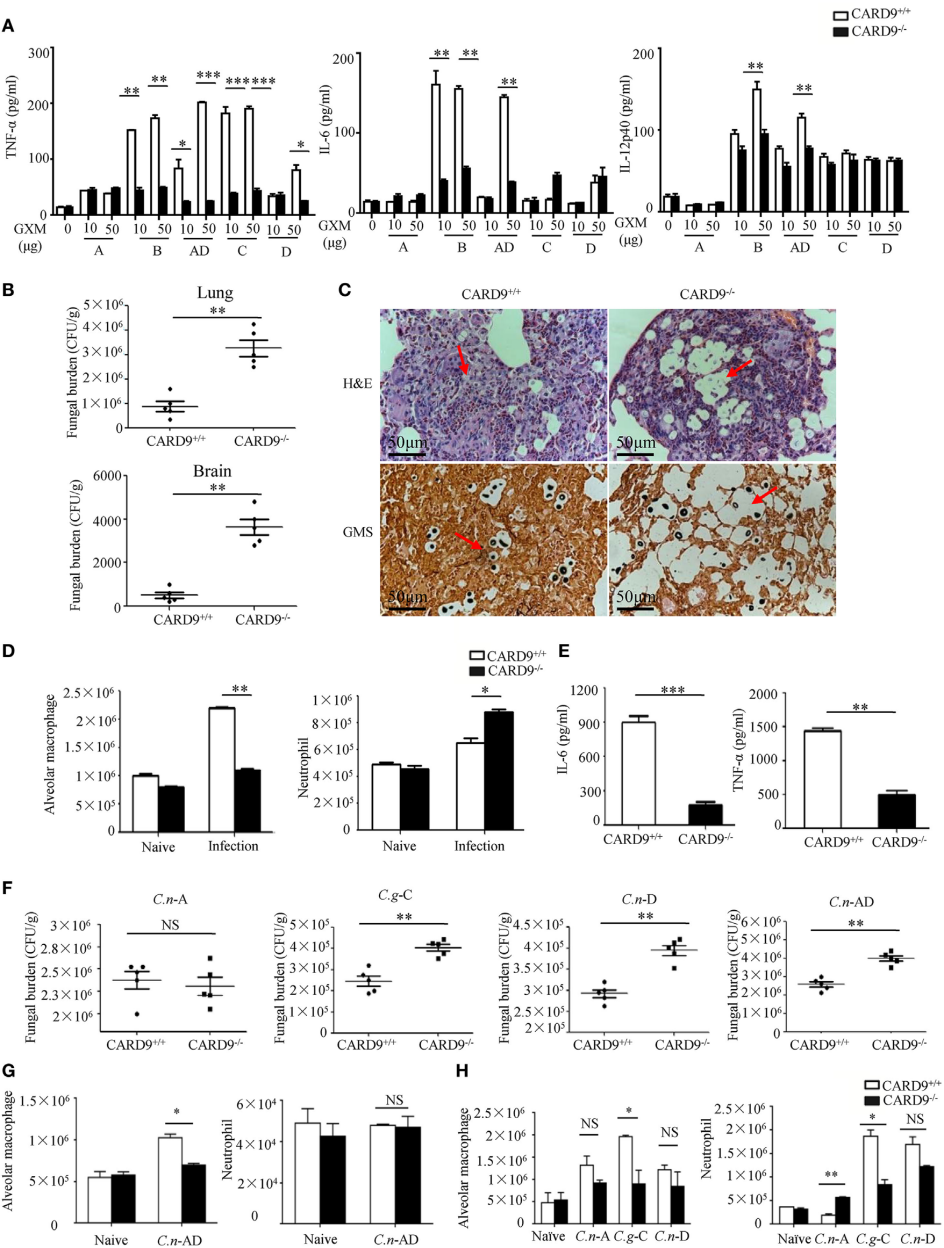
Caspase recruitment domain family member 9 (CARD9) is critical for pro-inflammation responses induced by GXMs. **(A)** Enzyme-linked immune-sorbent assay (ELISA) results for indicated cytokines in wild-type (WT) and CARD9-deficient bone marrow-derived macrophages (BMDMs), stimulated with plate-coated GXM extracted from *C.n-*A strain H99, *C.g-*B strain ATCC32609, *C.n-*AD strain WM628, *C.g-*C strain NIH312, and *C.n-*D strain WM629 for 16 h. **(B)** CFU assays of lung and brain of WT and CARD9-deficient mice after intratracheal infection with 1 × 10^5^ CFU of *C.g-*B strain ATCC32609 on day 3 post infection. **(C)** Histopathology with hematoxylin & eosin and Gomori’s methenamine silver staining on day 14 after intratracheal infection with 1 × 10^6^ CFU of *C.g-*B strain ATCC32609. **(D)** Alveolar macrophages (AMs) (CD11c^+^SiglecF^+^) and neutrophil (CD11b^+^Ly6G^+^) counts in lungs of WT and CARD9-deficient mice on day 1 after intratracheal infection with *C.g*-B strain ATCC32609. **(E)** ELISA results of TNF-a and IL-6 in lung homogenate of WT and CARD9-deficient mice on day 1 after infection. **(F)** Lung CFU assays of WT and CARD9-deficient mice on day 3 after intratracheal infection with 1 × 10^5^ CFU of *C.n-*AD strain WM628, *C.g-*B strain WM179, *C.g-*C strain NIH312, or *C.n-*D strain WM629, respectively. **(G,H)** AM (CD11c^+^SiglecF^+^) and neutrophil (CD11b^+^Ly6G^+^) counts in lungs of WT and CARD9-deficient mice on day 1 after intratracheal infection with *C.n-*AD strain WM628 **(G)** or *C.n-*A strain H99, *C.g-*C strain NIH312, and *C.n-*D strain WM629 **(H)**. Data are means ± SD of triplicate wells and are representative of three independent experiments; *p < 0.05, **p < 0.01, and ***p < 0.001.

It has been shown that CARD9 operates downstream of CLRs for activating NF-κB and ERK pathways (26, 27). To explore whether CARD9 is required for *C.g*-B-induced pro-inflammation responses, we stimulated BMDMs from WT and CARD9-deficient mice with plate-coated GXM extracted from *C.g-*B (GXM-B), and found that CARD9 deficiency in BMDMs completely impaired GXM-B induced activation of NF-κB and ERK pathways (Figures S4A,B in Supplementary Material). Furthermore, we found that CARD9 deficiency in BMDMs significantly impaired GXM-B induced secretion of pro-inflammation cytokines, including TNF-α and IL-6, but not IL12-p40 and IL-1β (Figure 7A). Moreover, CARD9 deficiency in BMDMs also significantly impaired the production of TNF-α and IL-6 when stimulated with plate-coated GXM extracted from *C.n*-AD, *C.g*-C, and *C.n*-D, but not *C.n*-A (Figure 7A). These data suggest that CARD9 is essential for NF-κB and ERK-mediated pro-inflammation responses induced by GXM from *C.g*-B, *C.g*-C, *C.n*-D, and *C.n*-AD.

To examine the effect of CARD9 deficiency on the clinical course of cryptococcal infection, we intratracheally infected WT and CARD9-deficient mice with different *Cryptococcus* strains. We found that the burdens in lung and brain were significantly higher in CARD9-deficient mice than those in WT mice on day 3 and 14 after infection with *C.g*-B strain ATCC32609 (Figure 7B; Figure S4C in Supplementary Material). Furthermore, the histological analysis of the lungs on day 14 after infection showed massive multiplication of yeast cells with poor granulomatous responses in the alveolar spaces of CARD9-deficient mice (Figure 7C), which is consistent with the findings observed in infected Dectin-3-deficient mice with *C.g*-B strain ATCC32609 (Figure 5C). Moreover, CARD9 deficiency completely blocked AM accumulation (Figure 7D; Figure S4D in Supplementary Material) and significantly reduced secretion of pro-inflammatory cytokine including TNF-α and IL-6 in the lungs on day 1 after infection with *C.g*-B (Figure 7E). Unexpectedly, CARD9 deficiency slightly increased neutrophil accumulation in the lungs (Figure 7D; Figure S4E in Supplementary Material), which has been reported in a previous study (28). Moreover, CARD9-deficient mice exhibited higher fungal burdens in lungs than WT mice after intratracheal infection with *C.n-*AD strain WM628, *C.g-*C strain NIH312, and *C.n-*D strain WM629 for 3 days (*p* < 0.01, Figure 7F). However, there were no differences in lung fungal burdens between WT- and CARD9-deficient mice when infected with *C.n-*A strain H99 on day 3 (Figure 7F). Moreover, CARD9 deficiency completely blocked AM accumulation in the lungs at day 1 after infection with *C.n-*AD strain WM628 and *C.g-*C strain NIH312 (Figures 7G,H; Figures S5 and S6 in Supplementary Material). Unexpectedly, CARD9 deficiency had no influences on AM accumulation in the lungs at day 1 after infection with *C.n-*D strain WM629 (Figure 7H; Figure S6 in Supplementary Material). These data show that CARD9 is critical for accumulation and activities of AM against pulmonary infection with *C.g*-B, *C.g-*C, and *C.n-*AD.

## Discussion

Germline encoded PRRs recognize a variety of microbial moieties which, once engaged, result in the activation of anti-microbial host defense and stimulation of adaptive immune responses. The role of CLRs during cryptococcosis is of interest as recent studies have defined their role in the recognition of carbohydrate moieties and host defense against other fungal pathogens (15, 16, 18, 21, 23, 42). In this study, we have extensively characterized the role of Dectin-3 and CARD9 during pulmonary cryptococcosis using an experimental murine model of pulmonary infection with *C. neoformans* and *C. gattii*. Upon pulmonary *C.n*-AD and *C.g*-B infection, Dectin-3- and CARD9-deficient mice were highly susceptible and showed augmented lung injury due to impairment of AM accumulation and killing activities, and subsequent productions of pro-inflammation cytokines and chemokines. More importantly, we showed that Dectin-3 directly recognized GXMs from *C.n*-AD and *C.g*-B to activate CARD9-mediated NF-κB and ERK pathways for initiating anti-fungal host responses against *C. neoformans* and *C. gattii*. Thus, our study provides the first biological and genetic evidence demonstrating that Dectin-3 recognizes GXM of *C. neoformans* serotype AD and *C. gattii* serotype B to initiate host defense against cryptococcosis.

However, there are still some intriguing parts in our study that worth further exploration. There has been over 120 years since the first isolation of *Cryptococcus* (43). Over several decades, *Cryptococcus* has been classified into two varieties including five serotypes: *C. neoformans* for serotypes A, D, and AD, and *C. gattii* for serotypes B and C (44). Furthermore, with the rapid development of PCR and DNA sequencing, these species have been further sub-divided into several genotypes: VNI, VII, VNB, VNIV, and VGI-IV (45). In this study, we used two different strains of *C. gattii* serotype B (ATCC32609 and WM179). Although they are the same serotype, the genotype of ATCC32609 (VGII) is different from WM179 (VGI). That could explain why the cytokine level of IL-12p40 stimulated by ATCC32609 was much lower than WM179. We assume that different genotypes may alter immunological property of *Cryptococcus* strains. In addition to serotypes, whether Dectin-3 can recognize different genotypes of *Cryptococcus* still needs further study.

*Cryptococcus* strains possess the large polysaccharide capsule to shield its cell wall components and the capsule is primarily composed of GXM, which comprises more than 90% of the capsular polysaccharide mass (29). In this study, both FACS screening and ELISA binding assay showed the direct binding of Dectin-3-Fc fusion protein with all five serotypes of GXM from *Cryptococcus* capsule. It was also confirmed that the general repeating unit composed of mannan and GluA determines its binding with Dectin-3. Although Dectin-3 can bind all five serotypes of GXM, only GXM-B and GXM-AD are dependent of Dectin-3 recognition to initiate pro-inflammatory responses. There are some possible explanations of this phenomenon. One is that the different quantity and position of xylose may alter the virulence of GXM and the disposition of the *O*-acetyl substituent may determine the antigenic activity (Figure S2E in Supplementary Material). Another reason is that not only Dectin-3, but also other PRRs can recognize *Cryptococcus* to initiate cell host responses (36).

In pulmonary cryptococcosis model, Dectin-3 deficiency significantly impaired accumulation and killing activity of AMs in lungs. In the meanwhile, Dectin-3-deficient mice had increased fungal burden in lungs. Adoptive transfer has been well established and applied to study the function of specific group of cells (46). In this study, adoptive transfer of WT AMs into Dectin-3-deficient mice rescued the number and percentage of AMs in lungs during *C.g*-B and *C.n*-AD infection. In consequence, the pro-inflammatory cytokine production increased and fugal burden was decreased. These data indicate that AM plays critical role in clearance of *Cryptococcus* infection. Complementary to our results, the depletion of AMs prior to infection results in rapid clinical deterioration and death of mice post *Cryptococcus* infection (47).

It is well-known that CARD9 is an adaptor molecule that plays critical role in anti-fungal immunity and is triggered through CLRs (28) In the present study, CARD9 deficiency impaired clearance of *C.g*-B, *C.g*-C, *C.n*-D, and *C.n*-AD whereas only *C.g*-B and *C.n*-AD are dependent of Dectin-3 recognition, which indicates that should be other CLRs that can recognize *C.g*-C and *C.n*-D. Dectin-2 is postulated to induce Th2-type responses and IL-4-dependent mucin production in the lungs following infection with *C.n-*D (20). The impact of Dectin-2 deficiency may also vary depending on the *Cryptococcus* serotypes.

As it is reported before, CARD9 is also important for recruitment of neutrophils to protect against invasive fungal infection (48). However, the impact of CARD9 on neutrophils recruitment and function against cryptococcal infection seems to be complicated depending on different serotypes of *Cryptococcus* infection. Besides, DCs are also critical for protection against pulmonary *Cryptococcus* infection. A recent study reported that plasmacytoid DCs have direct activity against *C. neoformans* requiring Dectin-3 expression and reactive oxygen species (49). Hence, whether different types of immune cells require Dectin-3 or CARD9 expression to initiate anti-cryptococcal effect deserves further study.

## Materials and Methods

### *Cryptococcus* Strain

*Cryptococcus gattii-*B strain ATCC32609 and WM179, *C. gattii-*C strain NIH312, *C. neoformans-*A strain H99, *C. neoformans-*D strain WM629, and *C. neoformans-*AD strain WM628 were gifted from Shanghai institute of fungal medicine, China. The yeast cells were cultured on Sabouraud dextrose agar (SDA) plates before use. Strains cultured in Yeast extract peptone dextrose (YPD) medium at 30°C for 16 h, were presented as thin-capsule form. Strains cultured in RPMI-1640 medium plus 10% FBS at 37°C with 5% CO_2_ for 16 h, were presented as thick-capsule form.

### BMDM Preparation

Primary cultures of BMDMs from C57B/L6 mice were prepared as previously described (50). Briefly, bone marrow cells were harvested from the femurs and tibias of mice. Erythrocytes were removed from cells by using a hypotonic solution. Cells were cultured for 7 days in DMEM medium containing 20% fetal bovine serum, 50 mM β-mercaptoethanol, 100 µg/mL penicillin–streptomycin, and 30% conditioned medium from L929 cells expressing M-CSF. On day 3, another 10 mL of the same medium was added. On day 7, non-adherent cells were removed and adherent cells were harvested.

### Analysis of Chemokine Genes Expression

Total RNA was extracted from the infected lungs or BMDMs using Triozl (TaKaRa Bio, Otsu, Japan), and the first-strand cDNA was synthesized using PrimeScript first-strand cDNAsynthesis kit (TaKaRa Bio, Otsu, Japan), according to the manufacturer’s instructions. Quantitative real-time PCR was performed in a volume of 20 µL using gene-specific primers and SYBR Premix Ex TaqII (TaKaRa Bio, Otsu, Japan) in a ABI 7500 system (Applied Biosystems, USA). The amounts of transcript were normalized to GAPDH. ΔΔCt method to calculate fold changes. First, Ct target gene − Ct housekeeping gene = ΔCt. Second, ΔCt treatment − ΔCt control = ΔΔCt. Third, fold changes = 2^−ΔΔCt^.

### Cytokine Assay

TNF-a, IL-6, IL-12p40, and IL-1β concentrations in the lung homogenates and culture supernatants were measured by SET-Ready-GO ELISA kits (eBioscience) according to the manufacturer’s protocol.

### Immunoblot Analysis

Harvest and pellet BMDM cells. Resuspend cell pellet in 250 µL ice cold lysis buffer (10 mM Hepes pH 7.9 + 10 mM KCl + 0.1 mM EDTA + 0.4% Nonidet P40) with protease inhibitors (Roche, 1836145) and incubate on ice for 15 min. Spin the lysate for 1 min at full speed at 4°C. Transfer supernatant to a fresh tube to keep the cytoplasmic fraction. Wash the nuclear pellet in 500 µL ice cold lysis buffer and spin down for 1 min at 4°C. Add 20 µL extraction buffer (20 mM Hepes pH 7.9 + 0.4 M NaCl + 1 mM EDTA) with inhibitors to the pellet and shake vigorously for 15 min on vortex in cold room. Spin for 10 min at full speed at 4°C. Transfer supernatant to fresh tube and store this nuclear extract at −20°C. Denature protein before use. Immunoblotting was performed as described previously (27). Anti-IκBα (9247), -phospho-IκBα (9246), -ERK (4372), -phospho-ERK (4695), and -NF-κB-p65 (8242) antibodies were obtained from Cell Signaling (Danvers, MA, USA). Anti-β-actin (ab8224) and -PCNA (ab152112) antibodies were from Abcam (Cambridge, MA, USA).

### Isolation and Purification of GXM

The extracted EPS from *Cryptococcus* strains were isolated as previously described (51). In detail, strains were grown in YNB culture medium at 30°C with shaking for 4 days and then the supernatant were collected after centrifuging with 10,000 × *g* for 5 min. The supernatant was slowly added with 3 volumes of EtOH and incubated for overnight at 4°C, which were centrifuged at 10,000 × *g* for 10 min. The precipitate was collected for air dry and then dissolved into 18 mL ddH_2_O. The polysaccharide concentration was determined by the phenol sulfuric method of Dubois (data not shown). To purify GXM, the polysaccharide solution was adjusted to 0.2 M NaCl and finalized with 3 times (w/w) of 0.3% (w/v) CTAB solution at room temperature, the mixture of which is named as CTAB-GXM. Then, CTAB-GXM was centrifuged at 10,000 × *g* for 10 min and the precipitate of CTAB-GXM was washed with 10% EtOH in ddH20 (v/v). To remove CTAB, the precipitate of CTAB-GXM was dissolved in 1 M NaCl and finalized with about 2.5 times (v/v) of EtOH with stirring. The precipitate containing GXM was collected after centrifuging at 10,000 × *g* for 10 min at room temperature and then dissolved in 2M NaCl until a viscous solution form. The viscous solution was dialyzed in a dialysis cassette with a 10,000MW cutoff for overnight against 1M NaCl and then against fresh ddH_2_O for 1 week. Finally, the solution EPS containing GXM inside the dialysis bag was lyophilized at −55°C for overnight. Endotoxin levels of GXM were measured by endotoxin assay kit and they were all within the detection range of 0.01–1 EU/mL (Figure S2D in Supplementary Material) (GenScript, Cat. No. L00350C).

### Analysis of Monosaccharide Composition of GXM

Extracellular polysaccharides (1 mg) was hydrolyzed with 2M TFA at 120°C for 2 h. After repeated evaporation with methanol to completely remove TFA, the residue was dissolved in distilled water and reduced with NaBH_4_ at room temperature for 3 h. After neutralization with AcOH and evaporation to dryness, the residue was acetylated with Ac_2_O for 1 h at 100°C. The resulting alditol acetates were analyzed with gas chromatography–mass spectrometry (GC–MS). The GC–MS temperature program for monosaccharide analysis was as follows: 140–198°C at 2°C/min, maintained for 4 min and increased to 214°C at 4°C/min, followed by increases of 1°C/min until 217°C was reached, which was maintained for 4 min and finally increased to 250°C at 3°C/min, maintained for 5 min as reported previously (52).

### Chemical Modification of GXM

Extracellular polysaccharides were performed with 1-cyclohexyl-3-(2-morpholinoethyl) carbodiimide metho-*p*-toluenesulfonate (CMC) and NaBH4 for carboxyl reduction (53). This procedure resulted in d- GluA converted into d-glucose. For deacylation, EPS (25 mg) was treated with 5 mL NaOH (0.1 M) for 16 h at 37°C, neutralized with 0.1 M HCl, and then dialyzed against water (54).

### Nuclear Magnetic Resonance (NMR) Analysis

Extracellular polysaccharides (80 mg) were dissolved in 0.4 mL D_2_O (99.8 Atom% Deuterium; Schweres Wasser, USA). The ^1^H NMR spectra were measured using the Bruker Avance III 600 Spectrometer (Bruker Instruments, Inc., Billerica, MA, USA) at 25°C. The chemical shifts of ^1^H NMR are expressed in ppm by using acetone as an internal standard; 4.70 ppm for ^1^H NMR. All the experiments were recorded, and data were processed using standard Bruker software and MestReNova (55).

### Isolation and Purification of CPS

The CPS from *Cryptococcus* strains were isolated as previously described (56). Briefly, *Cryptococcus* was recovered from 40% glycerin in −80°C and cultured in YPD (1% yeast extract, 2% peptone, 2% d-glucose, and 0.02% chloramphenicol) for 18 h. Then, yeast cells were washed three times with sterile PBS and resuspended in RPMI-1640 medium containing 10% FBS to induce thick capsule in cell incubator for 7 days. After that, yeast cells were collected and suspended in 15 ml of DMSO, incubating for 30 min twice. Cells were removed by centrifugation at 10,000 × *g* for 10 min, and the supernatant was dialyzed against by flowing ddH_2_O for 1 day. Finally, the CPS solution inside the dialysis bag was lyophilized.

### Plate-Coated GXM

For cell stimulation, GXM was dissolved in 70% ethanol at concentration of 500 µg/mL. Add 100 µL per well to 48-well cell culture plate and allow ethanol to volatilize, so that GXM was coated on plate. For ELISA binding assay, first, GXM was dissolved in PBS to form soluble polysaccharide solution at concentration of 10 µg/mL. Then, add 100 µL of GXM solution per well to 96-well ELISA plate and incubate overnight at room temperature. Finally, aspirate and wash three times with PBS.

### Expression of Fusion Protein

The extracellular domain of hDectin-3 was amplified from human PBMC cDNA. The primers were as follows: F (5′-3′): AGTCTTGCACTTGTCACGAATTCGTGTTTGGTGACTCATCACAA; R (5′-3′): GCATGTGTGAGT TTTGTCAGATCTGTTCAATGTTGTTCCAGGTA. The PCR program was: 95°C 2 min, 95°C 20 s, 58°C 20 s, 72°C 20 s, 40 cycles, 72°C 5 min, and 4°C hold. Then, PCR production was purified with PCR Clean Up Kit (AXYGEN). Meanwhile, pFUSE-hIgG1-Fc2 (InvivoGen, USA) were digested by EcoRI (Thermo Fisher, USA) and BglII (Thermo Fisher, USA) about 3 h at 37°C, the digesting production was purified with PCR Clean Up Kit. The gene that encoded the Dectin-3 extracellular domains was cloned into pFUSE-hIgG1-Fc2 expression vector. Herein, we used homologous recombination enzyme (HanBio, China) to ligate the plasmid and PCR production. The ligated plasmid was transformed into DH5α (Transgen, China). Select 3–5 bacterial colonies to culture in LB including zeocin (invivogen, USA) over night, the plasmid was obtained according to the manufacture of TIANpure MiNi Plasmid Kit (TINAGEN, China). Finally, CRD-hCLRs-pFUSE-hIgG1-Fc2 were sequenced by company (Huagene, China).

The hCLRs-pFUSE-hIgG1-Fc vectors were then transfected into 293T using Lipo2000 (Sigma), 1 day later, adding bleomycin (InvivoGen), the best proportion of bleomycin VS. DMEM is 3:1,000. After culturing 3 days, the cell supernatant was collected and the expression of CLR-Fc fusion protein was determined by immunoblot analysis with anti-Fc antibody.

### Fluorescence-Activated Cell-Sorting

Yeast cells underwent lag phase induction and were plated at 1 × 10^7^ cells/tube. Staining was performed in 0.5% BSA in PBS. Fusion proteins were then applied at 500 µL/tube followed by anti-human FITC-Fc secondary antibody (Abcam, Cambridge, MA, USA). Median fluorescence intensity of each tube was measured by BD FACS Array flow cytometer (BD Biosciences, San Jose, CA, USA) and presented as histogram.

### ELISA Binding Assay

Soluble GXM was adsorbed to ELISA plate at the concentration of 10 µg/mL. Then, plate was blocked with 5% nonfat milk at room temperature for 1 h. Meanwhile, fusion proteins were incubated separately with PBS/mannan/xylose/ GluA (final concentration: 1% solution) for 30 min at 37°C. Then, fusion proteins were added with 100 µL/well, incubated at 37°C for 2 h, and followed by 100 µL/well anti-human HRP-Fc secondary antibody (Jackson ImmunoResearch Laboratories, West Grove, PA, USA) at room temperature for 30 min. There were three times of washing between each step. After that, 100 µL/well substrates were added at room temperature for 10 min. Finally, 50 µL/well 1M phosphoric acid was added as stop solution. Test results were measured by plate reader at 450 nm.

### Mice

Wil-type (WT) C57BL/6, Dectin-3-deficient (Clec4d*^−/−^*) and CARD9-deficient (CARD9*^−/−^*) mice were kept under specific-pathogen-free conditions at the Institute for Animal Experimentation, Tongji University School of Medicine. 6- to 8-week-old male and female mice were used in this study. No evidence of susceptibility based on sex was observed.

### Pulmonary Cryptococcal Infection in Mice

Mice were anesthetized by inhaling isoflurane (RWD life science, Shenzhen, China) and restrained on a small board. Live yeast cells (1 × 10^5^ or 1 × 10^6^) were inoculated in a 35 µL volume into the trachea of each mouse.

### Fungal Burden Analysis

Mice were sacrificed on day 3 after infection, and lungs or brains were dissected carefully, excised, and then homogenized separately in 1 ml of distilled water by teasing with a stainless mesh at room temperature. The homogenates, diluted appropriately with distilled water, were inoculated at 100 µL on SDA plates and cultured for 2 days, and the resulting colonies were counted.

### Histological Examination

The lung specimens obtained from mice were fixed in 10% buffer formalin, dehydrated, and embedded in paraffin. Sections were cut and stained with hematoxylin & eosin or Gomori’s methenamine silver stain, using standard staining procedures, at pathology platform of Servicebio Technology, Wuhan, China.

### Flow Cytometry and Cell Sorting

Anti-mouse antibodies to CD45-FITC, CD11b-PerCP-Cy5.5, CD11c-APC, Ly6G-BV421, Siglec-F-PE, and fixable viability stain 780 were obtained from BD Pharmingen. The lungs from mice were perfused and digested into single-cell suspensions as described previously (57). After RBC lysis buffer treatment, the whole lung cells were washed with PBS and then stained with corresponding fluorescent antibodies. Following incubation, samples were washed and fixed in 2% ultrapure formaldehyde. The absolute number of each leukocyte subset was then determined by multiplying the absolute number of CD45^+^ cells by the percentage of cells stained by fluorochrome-labeled antibodies for each cell population analyzed using BD FACSArray software™ on a BD FACSArray flow cytometer (BD Biosciences, San Jose, CA, USA). AMs were identified as CD45^+^CD11c^+^Siglec-F^+^. Neutrophils were identified as CD45^+^CD11b^+^Ly6G^+^.

Cell sorting was performed on BD FACSAria II instrument, using BD FACSDiva software (BD Biosciences), and compensation and data analyses were performed using FlowJo software (TreeStar, Ashland, OR, USA). Cell populations were identified using sequential gating strategy. The sorting purity was 90–95%.

### *In Vitro* Killing Assay

Alveolar macrophages were sorted from the lung leukocytes of mice. The purified AMs (1 × 10^4^/well) were cultured, in triplicate, within individual wells of a 96-well U-bottom tissue culture plate in DMEM complete media containing *C.n*-A, *C.g*-B, and *C.n*-AD respectively at multiplicity of infection of 1, 0.1, and 0.01; and with *C.n*-A, *C.g*-B, and *C.n*-AD in DMEM complete media without AMs as a control. After 6 h, the content of each well was centrifuged and the supernatants were removed. The AMs in the cell pellet were then lysed by washing three times with sterile water and incubating in water for 20 min. The remaining yeast was diluted in PBS and plated onto SDA plate to quantify the live cryptococcal cells.

### Adoptive Transfer Experiments

For adoptive transfer experiments, AM (CD45^+^CD11c^+^siglec-F^+^) from WT and Dectin-3-deficient mice were sorted using a FACSAria II (BD Biosciences). Then freshly sorted AM (5 × 10^5^ cells in 200 µL PBS) were transferred intravenously into WT and Dectin-3-deficient mice as described previously (58). At 2 h after adoptive transfer, mice were challenged with 1 × 10^5^ CFU of *C.g-*B strain ATCC32609. At day 1 after challenge, mice were sacrificed and samples were collected for the following experiments.

### Statistical Analysis

At least two biological replicates were performed for all experiments unless otherwise indicated. Log-rank testing was used to evaluate the equality of survival curves. Student’s *t*-test for paired observations was used for statistical analyses of cytokine expression levels. Statistical significance was set at a *P* value of less than 0.05, 0.01, or 0.001.

## Ethics Statement

All animal experimental procedures were performed in accordance with the Regulations for the Administration of Affairs Concerning Experimental Animals approved by the State Council of People’s Republic of China. The protocol was approved by the Institutional Animal Care and Use Committee of Tongji University (Permit Number: TJLAC-015-002). Human peripheral blood mononuclear cells (PBMCs) were obtained from Department of Medical Laboratory, Shanghai Pulmonary Hospital of China.

## Author Contributions

H-RH, J-FX, and X-MJ designed the experiments. H-RH, FL, HH, XX, NL, and SW performed the experiments. H-RH, J-FX, and X-MJ analyzed the data and wrote the manuscript with editorial input from all the authors.

## Conflict of Interest Statement

The authors declare that the research was conducted in the absence of any commercial or financial relationships that could be construed as a potential conflict of interest.
