# Supplementary figures and images for: Dectin-3 Recognizes Glucuronoxylomannan of Cryptococcus neoformans Serotype AD and Cryptococcus gattii Serotype B to Initiate Host Defense Against Cryptococcosis
Source: Front Immunol. 2018 Aug 6;9:1781. doi: 10.3389/fimmu.2018.01781 (PMC6090260; doi:10.3389/fimmu.2018.01781)

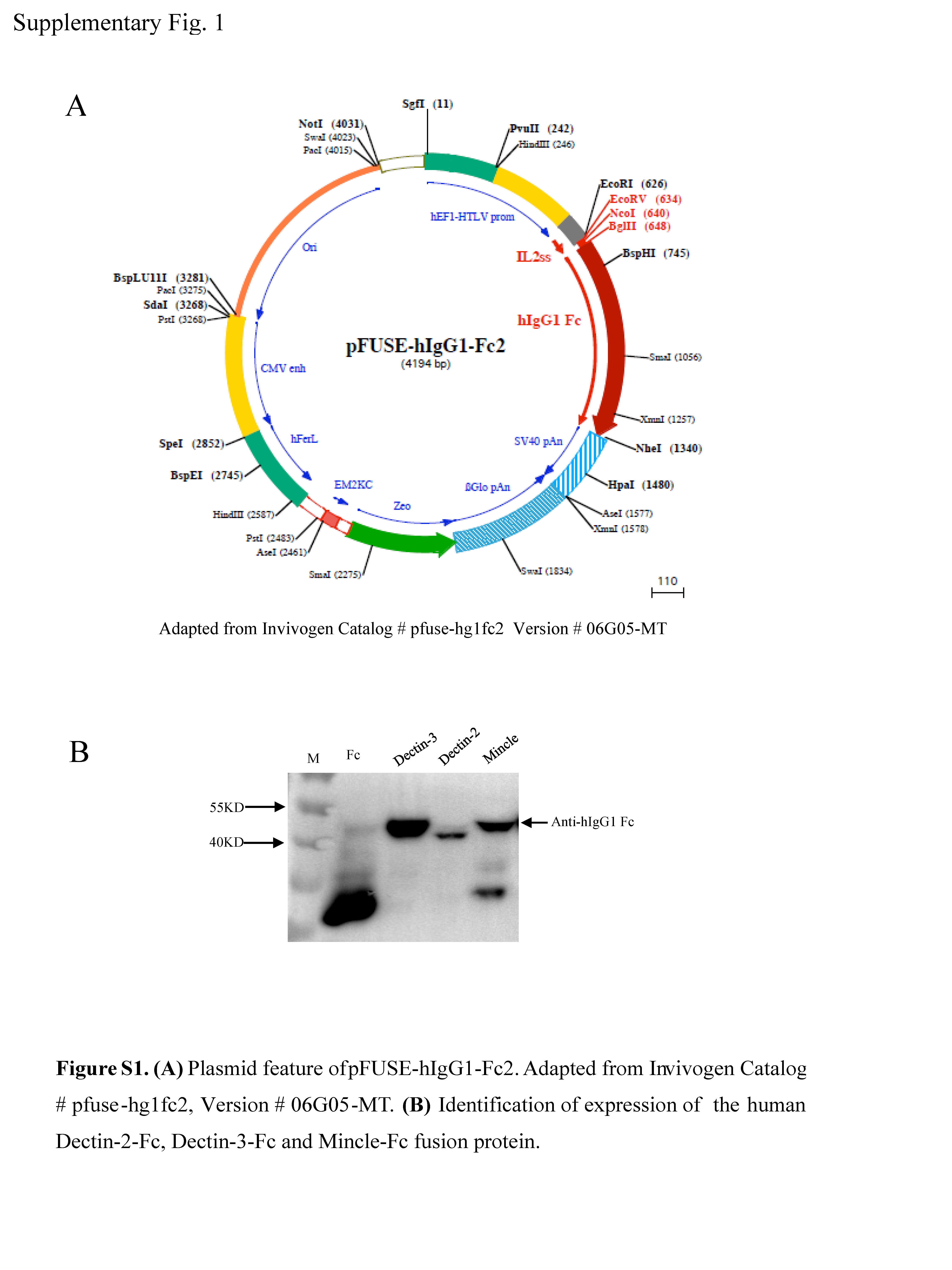

Supplement: Supplementary file 1 [file image_1.tiff]

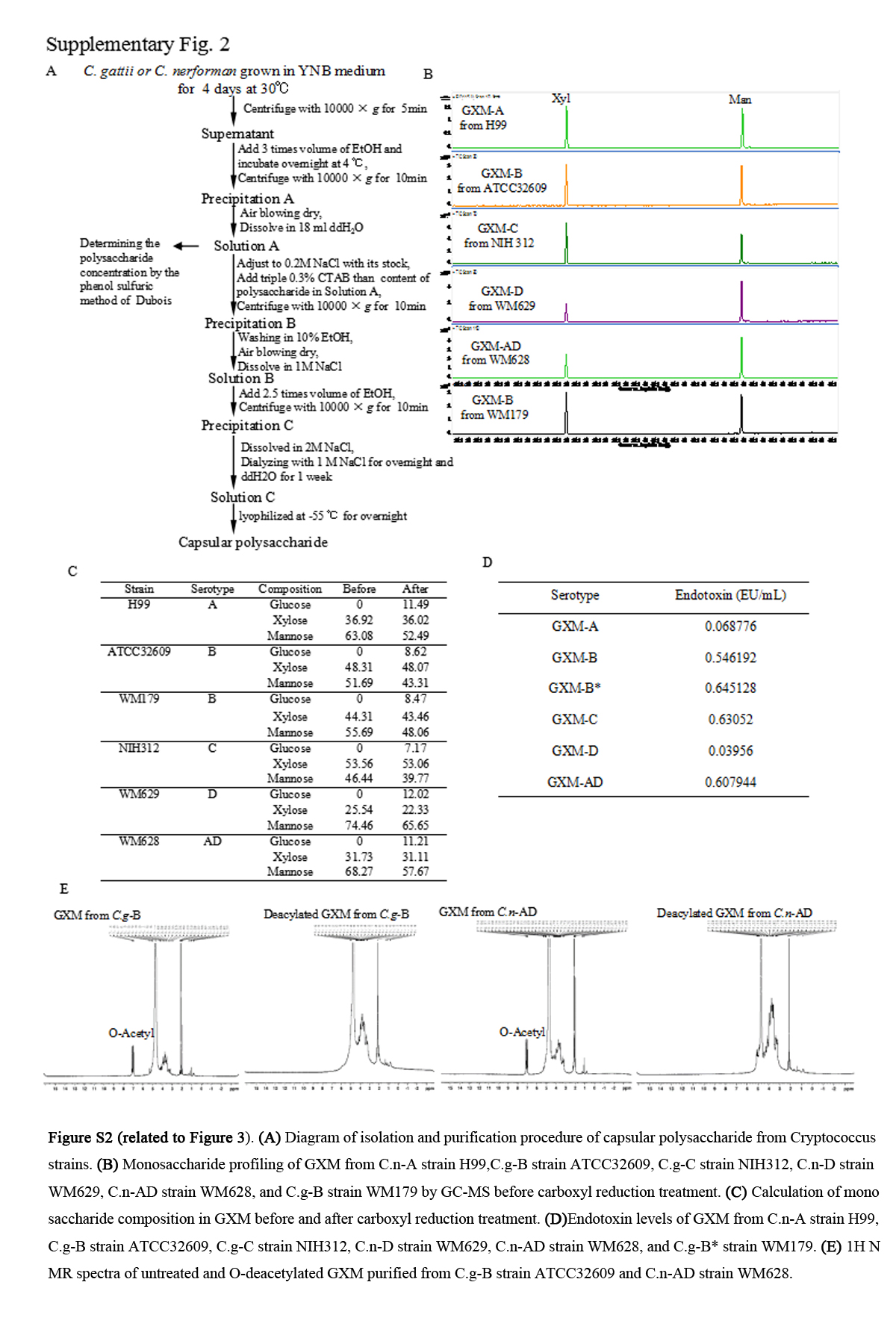

Supplement: Supplementary file 2 [file image_2.jpeg]

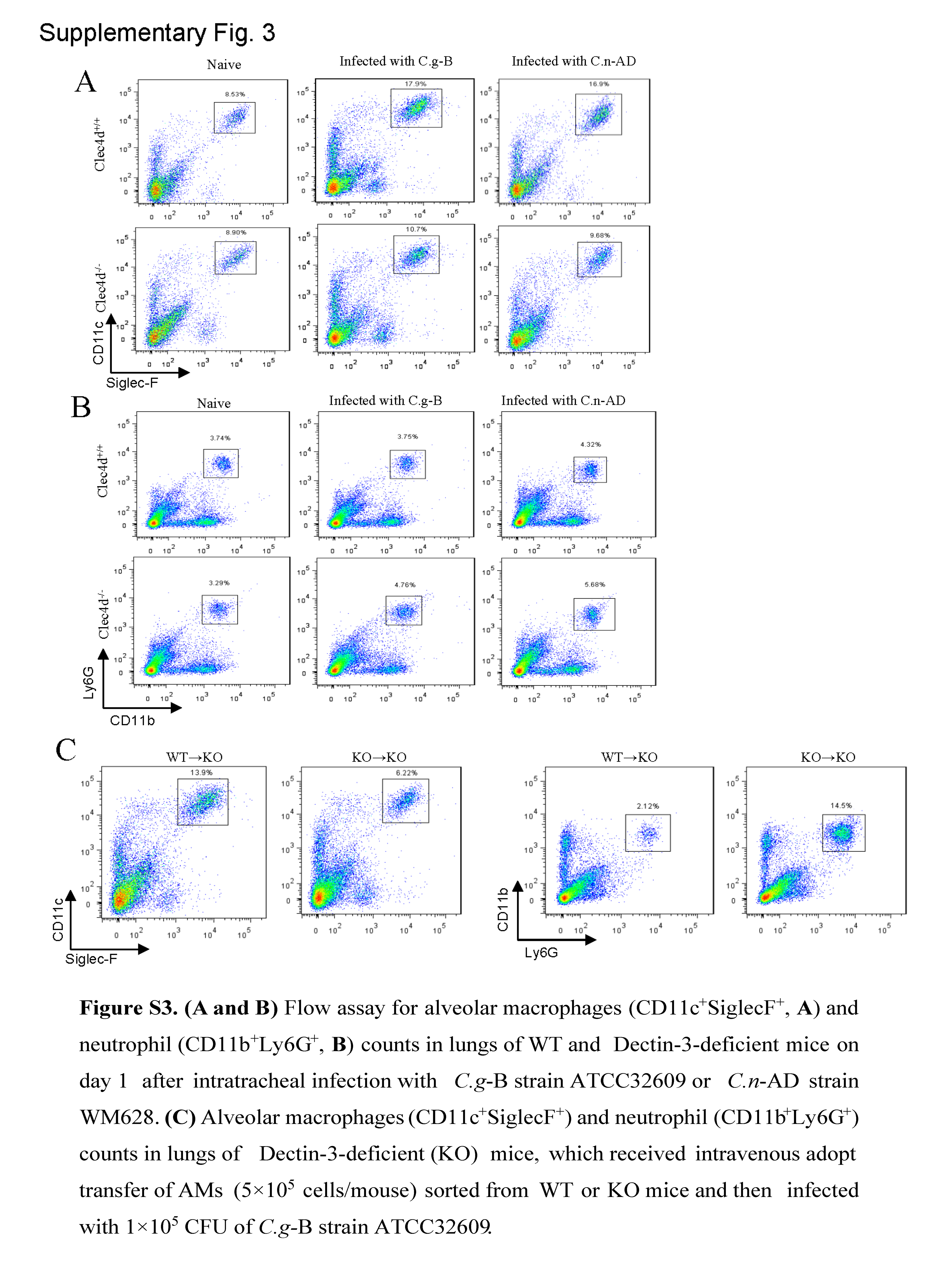

Supplement: Supplementary file 3 [file image_3.tiff]
