# Supplementary material for: Dectin-3 Recognizes Glucuronoxylomannan of Cryptococcus neoformans Serotype AD and Cryptococcus gattii Serotype B to Initiate Host Defense Against Cryptococcosis
Source: Front Immunol. 2018 Aug 6;9:1781. doi: 10.3389/fimmu.2018.01781 (PMC6090260; doi:10.3389/fimmu.2018.01781)
Supplement: Supplementary file 4 [file image_4.pdf]

Supplementary Fig. 4

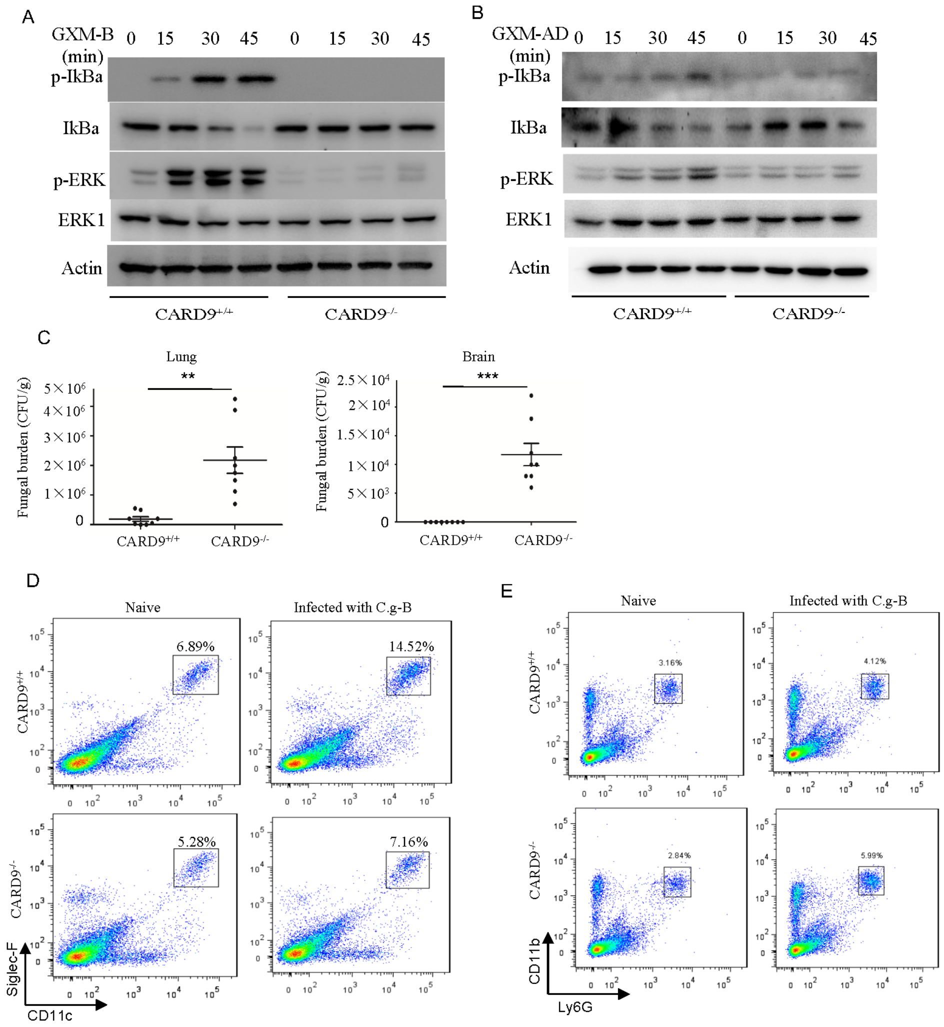

Figure S4. (A and B) Protein phosphorylation levels in BMDMs from WT and CARD9-deficient mice, which were stimulated with plate-coated 50μg/well GXM from *C.g*-B strain ATCC32609 (A) or *C.n*-AD strain WM628 (B) for indicated time. **(C)** CFU assays of lung and brain of WT and CARD9-deficient mice mice infected intratracheally with 1×10^6 CFU of *C.g*-B strain ATCC32609 on day 14 post infection. **p<0.01, ***p<0.001 **(D and E)** Flow assay for alveolar macrophages ($CD11c^+SiglecF^+$, **D**) and neutrophil ($CD11b^+Ly6G^+$, **E**) counts in lungs of WT and CARD9-deficient mice on day 1 after intratracheal infection with *C.g*-B strain ATCC32609.
