# Supplementary material for: Dectin-3 Recognizes Glucuronoxylomannan of Cryptococcus neoformans Serotype AD and Cryptococcus gattii Serotype B to Initiate Host Defense Against Cryptococcosis
Source: Front Immunol. 2018 Aug 6;9:1781. doi: 10.3389/fimmu.2018.01781 (PMC6090260; doi:10.3389/fimmu.2018.01781)
Supplement: Supplementary file 5 [file image_5.pdf]

Supplementary Fig. 5

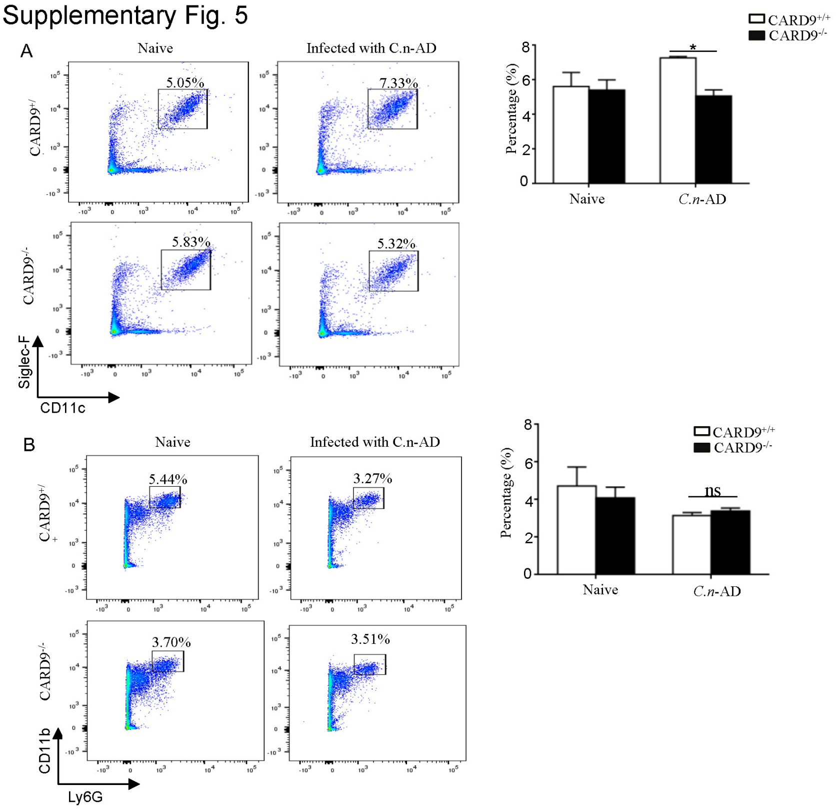


**Figure S5. (A and B)** Flow assay for alveolar macrophages (CD11c$^{+}$SiglecF$^{+}$, **A**) and neutrophil (CD11b$^{+}$Ly6G$^{+}$, **B**) counts in lungs of WT and CARD9-deficient mice on day 1 after intratracheal infection with *C.n*-AD strain WM628.
