# Supplementary material for: Dectin-3 Recognizes Glucuronoxylomannan of Cryptococcus neoformans Serotype AD and Cryptococcus gattii Serotype B to Initiate Host Defense Against Cryptococcosis
Source: Front Immunol. 2018 Aug 6;9:1781. doi: 10.3389/fimmu.2018.01781 (PMC6090260; doi:10.3389/fimmu.2018.01781)
Supplement: Supplementary file 6 [file image_6.pdf]

Supplementary Fig. 6

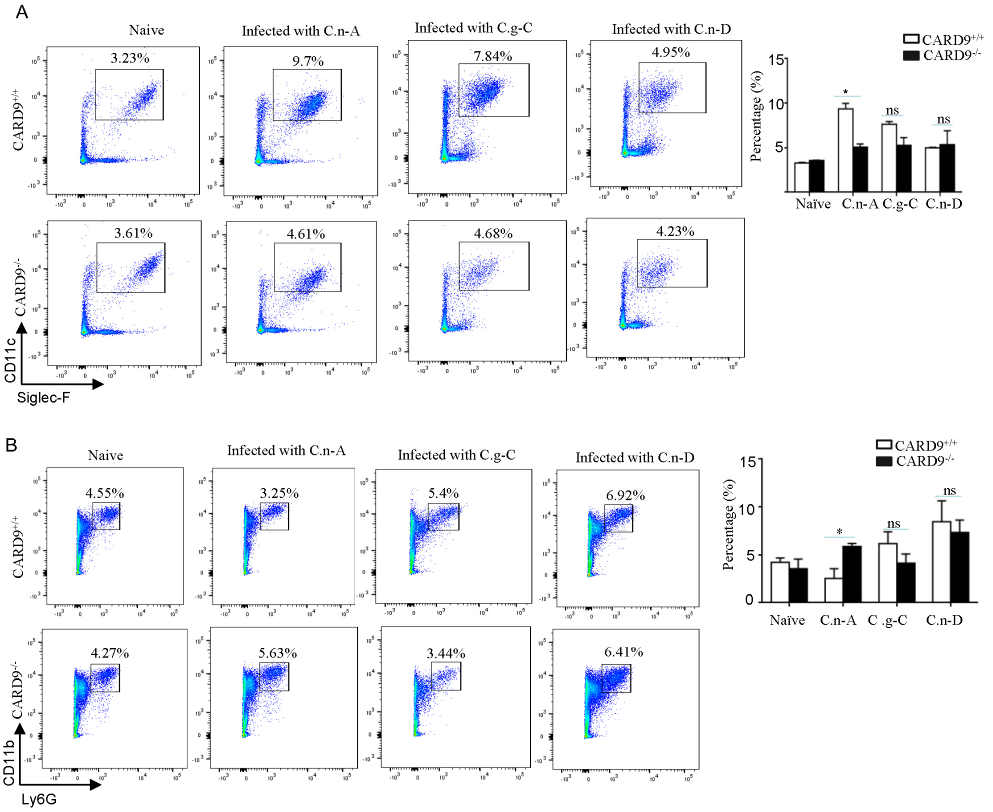

Figure S6. (A and B) Flow assay for alveolar macrophages (CD11c$^+$SiglecF$^+$, **A**) and neutrophil (CD11b$^+$Ly6G$^+$, **B**) counts in lungs of WT and CARD9-deficient mice on day 1 after intratracheal infection with *C.n*-A strain H99, *C.g*-C strain NIH312 and *C.n*-D strain WM629.
